# Proteomic profiling of *Juniperus oxycedrus* and *Cupressus arizonica* pollen reveals quantitative interspecies differences in the IgE-binding protein repertoire associated with Mediterranean Cupressaceae allergy

**DOI:** 10.3389/falgy.2026.1885294

**Published:** 2026-08-05

**Authors:** Sandra Sivill, Jose Carlos Hernández-Walias, Juan Manuel Igea, Jose Alejandro Lemus Calderon, Ana Maria Navarro-Pulido, Carmen Rondon, Beatriz Fernandez Parra, Magdalena Lluch-Bernal, M. Teresa Dordal Culla, Fernando Pineda, Jose Luis Subiza, Salvador Iborra, Jose F. Cantillo

**Affiliations:** 1 Inmunotek, S.L., R&D Department, Alcalá de Henares, Madrid, Spain; 2 Allergy Unit, Clínica Alergoasma, Salamanca, Spain; 3 Allergy Unit, Hospital General de Villarobledo, Albacete, Spain; 4 Allergy Unit, Hospital Universitario Virgen Macarena, Sevilla, Spain; 5 Allergy Unit, Hospital Regional Universitario de Malaga, Malaga, Spain; 6 Allergy Group. Instituto de Investigación Biomédica de Málaga y Plataforma en Nanomedicina - IBIMA Plataforma BIONAND. RICORS Inflammatory Diseases, Malaga, Spain; 7 Allergy Unit, Hospital Universitario El Bierzo, Ponferrada/León, Spain; 8 Allergy Unit, Hospital Universitario La Paz, Madrid, Spain; 9 Allergy Unit, Hospital Universitari de Bellvitge, L'Hospitalet de Llobregat, Barcelona, Spain

**Keywords:** allergen cross-reactivity, Cupressaceae allergy, *Cupressus arizonica*, IgE sensitization, *Juniperus oxycedrus*, pollen IgE-binding proteins, proteomics

## Abstract

**Background:**

Diagnosis and allergen-specific immunotherapy for Mediterranean allergy rely on extracts standardized to group-1 allergens, such as Cup a 1 from *Cupressus arizonica*, potentially overlooking interspecies variability. The native species *Juniperus oxycedrus*, widespread in Spain, may contribute to sensitization, but its clinical relevance and allergen repertoire remain poorly characterized.

**Objective:**

To characterize and compare the allergen and IgE-binding protein composition of *J. oxycedrus* and *C. arizonica* pollen extracts and assess their sensitizing relevance in Spain.

**Methods:**

Pollen extracts were analyzed using SDS-PAGE, two-dimensional electrophoresis (2-DE), Western blotting (WB), and mass spectrometry (MS). 241 patients with allergic rhinitis and/or conjunctivitis from seven Spanish regions underwent skin prick testing and specific IgE for extracts and Jun o 1/Cup a 1, by ELISA. Cross-reactivity was assessed by competitive ELISA and WB inhibition.

**Results:**

Proteomics and 2-DE showed a more complex protein profile in *J. oxycedrus* than in *C. arizonica* pollen extract (423 vs. 251 proteins, 146 shared). Among patients, 35% were sensitized to Cupressaceae, with more frequent monosensitization to *J. oxycedrus*. 2-DE/WB showed ≥60 IgE-binding spots in *J. oxycedrus* vs. 5 in *C. arizonica*, including group-1 allergen isoforms and IgE-reactive components (calreticulin, superoxide dismutase, aconitate hydratase, aldose 1-epimerase, and enolase) with lower abundance in *C. arizonica*. Inhibition assays indicated high but asymmetric cross-reactivity, consistent with the differences in IgE-binding protein abundance.

**Conclusion:**

*J. oxycedrus* and *C. arizonica* pollen extracts display broadly similar IgE-reactive repertoires but differ in the relative abundance. Such differences may affect regional sensitization and limit the effectiveness of group 1–based diagnosis and therapy.

## 1 Introduction

The pollen from Cupressaceae is an important risk factor for pollinosis, particularly in the Mediterranean region, and represent one of the most sensitizing allergen source (1). Globally, Cupressaceae allergy is a growing health burden (2), with sensitization rates of 10%–35% (2–5). Sensitization rates vary regionally, aligning with the distribution and flowering of Cupressaceae species (6). In Spain, where Cupressaceae trees are widespread (6), rates reach 35% in Córdoba (7) and 23% in Madrid (8), with similar patterns across the Mediterranean (4, 5).

In clinical practice, allergy to the pollen of Cupressaceae is diagnosed using extracts from *Cupressus arizonica* (currently classified as *Hesperocyparis arizonica*, although the traditional name is retained in the allergy literature and allergen nomenclature, e.g., Cup a 1), or related species containing group 1 pectate lyases (e.g., Cup a 1), despite the potential existence of multiple allergenic components (9, 10). This strategy is supported by the high IgE-binding frequency to Cup a 1 (11) and strong cross-reactivity among Cupressaceae species, attributed to the structural conservation of group 1 pectate lyases (11, 12). However, this approach assumes immunological equivalence across species, overlooking critical variables such as inter- and intra-species molecular variation in the allergen repertoire, abundance, and geographic distribution.

In Europe, *C. arizonica* is an introduced species widely planted in urban and peri-urban areas (13, 14), whereas *Juniperus oxycedrus* is native in Mediterranean woodlands (15, 16). Local populations inhale both pollen types, and sensitization may reflect distinct or overlapping responses. Previous work from our group identified and characterized the pectate lyase major allergen Jun o 1 from *J. oxycedrus*, demonstrating that, although structurally homologous to Cup a 1, it differs in its capacity to form oligomers and exists in variable molecular weights, which could provide additional IgE-binding determinants which are not present in Cup a 1 (17).

We hypothesized that the pollen of *J. oxycedrus* not only contains Jun o 1 but may also include components that appear less represented in *C. arizonica*. Together with potential differences in the relative abundance and oligomeric forms of Jun o 1, these factors could contribute to divergent sensitization profiles and may have implications for the performance of diagnostic tests and allergen-specific immunotherapy (AIT), which is currently based on group 1-standardized extracts. In this context, a more comprehensive characterization of *J. oxycedrus* allergen repertoire is warranted to assess whether reliance on Cup a 1 as a primary marker may lead to underestimation of clinically relevant sensitizations in Mediterranean populations. This is particularly relevant given that *J. oxycedrus* is native to the region, in contrast to *C. arizonica*, which has been introduced. The use of native species is advantageous, as it better reflects the historical and ongoing natural exposure of local populations, thereby providing a more realistic representation of sensitization patterns under real-life environmental conditions.

In this study, we conducted an integrated cross-sectional molecular analysis using sera from allergic individuals residing in seven regions of Spain. We assessed sensitization patterns to pollen extracts and major allergens of *J. oxycedrus* and *C. arizonica* and combined these findings with a comparative characterization of their IgE-binding repertoires. Using SDS-PAGE, two-dimensional electrophoresis, western blotting, and mass spectrometry, we profiled the protein composition and abundance of both extracts and evaluated differences in IgE-binding patterns between species.

## 2 Materials and methods

### 2.1 Pollen extracts and native allergens

Lyophilized and defatted *J. oxycedrus* and *C. arizonica* raw material were purchased from Iber-Polen (Spain) and extracted 1:10 overnight (ON) with Phosphate-Buffered Saline (PBS), pH 7.2 at 4 °C in constant stirring. After extraction, the mixture was centrifuged at 15,000 RCF for 10 min and the supernatant filtered through a 0.2 µm pore membrane (Sartorius Stedim Biotech). After filtration, the extract was dialyzed against milliQ water in 3,500 Da cut-off membranes (Spectra/Por), lyophilized and stored until usage. The protein concentration was determined by Bradford assay, using bovine serum albumin (BSA) as a standard (Bio-Rad). Jun o 1 and Cup a 1 were purified by affinity chromatography in a HiTrap™ Con A 4B column (Cytiva), using a binary gradient of binding buffer and elution buffer (20 mM Tris-HCl, 0.5 M NaCl, 0.5 M Methyl-α-D-glucopyranoside, pH 7.4), as described (17).

### 2.2 Characterization of pollen extracts by mass spectrometry

Protein and IgE-binding protein composition were determined by MS (18), conducted at the Proteomics Unit, National Center for Biotechnology (CNB-CSIC), Madrid, Spain. Protein extracts were processed using the S-Trap™ digestion workflow. Each sample was diluted with lysis buffer to 2.5% sodium dodecyl sulfate (SDS), 3M urea and 50 mM triethylammonium bicarbonate (TEAB). Samples were reduced and alkylated with 10 mM tris(2-carboxyethyl) phosphine (TCEP) and 10 mM chloroacetamide (CAA) for 1 h at 37 °C. Proteins were digested in the S-Trap filter (Protifi, Huntington, NY, USA) following the manufacturer's instructions with minor changes (19). Each sample was digested at 37 °C ON using a trypsin:protein ratio of 1:15 and was cleaned with a StageTip C18.

The resulting peptide mixtures were analyzed by Liquid Chromatography Electrospray Ionization Tandem Mass Spectrometric (LC-ESI-MS/MS). Peptide concentration was determined by Qubit™ Fluorometric Quantitation (Thermo Fisher Scientific). Samples (5 µL) diluted in 2% ACN in 0.1% FA, containing 500 ng of digested proteins, were subjected to 1D-nano LC ESI-MS/MS analysis using an Ultimate 3,000 nano HPLC system (Thermo Fisher Scientific) coupled online to an Orbitrap Exploris 240 mass spectrometer (Thermo Fisher Scientific). Peptides were eluted onto a 50 cm × 75 μm Easy-spray PepMap C18 analytical column at 45 °C and separated at 250 nL/min using a gradient from 2% to 95% in 60 min. The mobile phases used were: mobile phase A 0.1% formic acid (FA); mobile phase B: 100% acetonitrile (ACN), 0.1% FA.

Data acquisition was performed using a data-dependent top-20 method, in full scan positive mode, scanning 350–1,200 m/z. Survey scans were acquired at a resolution of 60,000 at m/z 200, with Normalized Automatic Gain Control (AGC) target (%) of 300 and a maximum injection time (IT) of 40 ms. The top 20 most intense ions from each MS1 scan were selected and fragmented via Higher-energy collisional dissociation (HCD), at a resolution set to 45,000 at m/z 200, with AGC target of 200 and maximum ion injection time of 120 ms. Isolation of precursors was performed with a window of 1 m/z, exclusion duration (s) of 45 and HCD collision energy of 32. Precursor ions with single, unassigned, or six and higher charge states from fragmentation selection were excluded.

The acquired spectra were used for proteomics data analysis and sequence searching. Raw instrument files were processed using Proteome Discoverer (PD) version 2.5.0.400 (Thermo Fisher Scientific). MS2 spectra were searched using Mascot Server v2.8.1 (Matrix Science, London, UK) against the Pinidae UniProtKB database (20251015, 142,477 sequences) containing the most common laboratory contaminants (cRAP database with 70 sequences). All searches were configured with dynamic modifications for pyrrolidone from Q (−17.027 Da) and oxidation of methionine residues (+15.9949 Da) and static modification as carbamidomethyl (+57.021 Da) on cysteine, monoisotopic masses, and trypsin cleavage (max 2 missed cleavages). The peptide precursor mass tolerance was 10 ppm, and MS/MS tolerance was 0.02 Da. The false discovery rate (FDR) for proteins, peptides, and peptide spectral matches (PSMs) peptides was set to 1%.

Precursor ion quantitation was performed in Proteome Discoverer using the “Minora” feature in the processing method and the “Feature Mapper” and “Precursor Ions Quantifier” nodes in the consensus step. Protein abundances were calculated by summing sample abundances of the connected peptide groups (using unique + razor peptides).

### 2.3 Study population

The study was conducted within the framework of the Precision allergic rhinitis: Multicentric MOLecular mapping of the allergic EXPOsome in Spain (EXPOMOL Study). Patients with a clinical diagnosis of allergic rhinitis and/or conjunctivitis were recruited from seven allergy centers from Spain: Salamanca, León, Sevilla, Málaga, Madrid, Albacete, and Barcelona (Table 1 and Supplementary Table S1). All participants underwent skin prick testing (SPT) using a standardized panel of aeroallergens, including *J. oxycedrus*, *C. arizonica* and *C. sempervirens*. Tests were performed according to established guidelines, and reactions were read after 15–20 min. A positive result was defined as a wheal diameter ≥3 mm greater than that of the negative control. The study was designed to characterize sensitization to Cupressaceae pollens in this population; however, the clinical relevance of individual Cupressaceae species was not specifically established. Serum samples were obtained after written informed consent and specific IgE to *J. oxycedrus* and *C. arizonica* extracts, and Jun o 1 and Cup a 1, was analyzed in the sera from individuals with positive SPT to at least one Cupressaceae species. Approval was obtained from: the Ethic Committees of drugs investigation of: Salamanca health area, Health service areas of León and El Bierzo and University Hospital Complex of the Canary Islands; the Research Ethics Committee on Medicinal Products of Integrated Healthcare Management of Albacete and Malaga; the Research Ethics Committee of Virgen Macarena—Virgen del Rocío University Hospitals of Seville and the Research Ethics Committee of the Bellvitge University Hospital.

**Table 1:** Demographic and Cupressaceae sensitization profiles in Spanish patients with allergic rhinoconjunctivitis.

Characteristic	Total (*n* = 241)	Salamanca (*n* = 45)	Albacete (*n* = 35)	León (*n* = 35)	Málaga (*n* = 45)	Sevilla (*n* = 28)	Madrid (*n* = 27)	Barcelona (*n* = 26)
Prevalence of Cupressaceae Sensitization by Skin Prick Test Positivity								
Male, *n* (%)	114 (47.3)	18 (40.0)	15 (42.9)	17 (48.6)	22 (48.9)	16 (57.1)	18 (66.7)	8 (30.8)
Age in years, mean ± SD	33.0 ± 15.2	35.3 ± 14.9	27.2 ± 12.2	26.8 ± 14.6	32.1 ± 15.0	36.3 ± 16.0	35.1 ± 11.6	40.6 ± 18.2
Skin Prick Test, *n* (%)								
Positive to ≥1 species	84 (34.9)	25 (55.6)	15 (42.9)	4 (11.4)	9 (20.0)	8 (28.6)	17 (63.0)	6 (23.1)
Positive to *J. oxycedrus*	74 (30.7)	22 (48.9)	15 (42.9)	3 (8.6)	7 (15.6)	7 (25.0)	16 (59.3)	4 (15.4)
Positive to *C. arizonica*	71 (29.5)	23 (51.1)	14 (40.0)	2 (5.7)	6 (13.3)	4 (14.3)	16 (59.3)	6 (23.1)
Positive to *C. sempervirens*	71 (29.5)	23 (51.1)	13 (37.1)	3 (8.6)	5 (11.1)	4 (14.3)	17 (63.0)	6 (23.1)
Monosensitized, *n* (%)^a^								
Only to *J. oxycedrus*	7 (8.3)	0 (0.0)	1 (6.7)	1 (25.0)	2 (22.2)	3 (37.5)	0 (0.0)	0 (0.0)
Only to *C. arizonica*	2 (2.4)	0 (0.0)	0 (0.0)	0 (0.0)	1 (11.1)	1 (12.5)	0 (0.0)	0 (0.0)
Only to *C. sempervirens*	3 (3.6)	1 (4.0)	0 (0.0)	1 (25.0)	1 (11.1)	0 (0.0)	0 (0.0)	0 (0.0)
Frequency of Cupressaceae-Specific IgE Among Patients with Positive Skin Prick Tests								
Specific IgE by ELISA, *n* (% within positive SPT to ≥ 1 species)								
Specific IgE to ≥1 species	73 (86.9)	23 (92.0)	14 (93.3)	2 (50.0)	6 (66.7)	7 (87.5)	16 (94.1)	5 (83.3)
Specific IgE to *J. oxycedrus*	55 (65.5)	16 (64.0)	12 (80.0)	2 (50.0)	4 (44.4)	6 (75.0)	14 (82.4)	1 (16.7)
Specific IgE to *C. arizonica*	69 (82.1)	21 (84.0)	14 (93.3)	2 (50.0)	6 (66.7)	7 (87.5)	15 (88.2)	4 (66.7)
Specific IgE Jun o 1	67 (79.8)	25 (100.0)	13 (86.7)	2 (50.0)	6 (66.7)	6 (75.0)	15 (88.2)	4 (66.7)
Specific IgE to Cup a 1	60 (71.4)	19 (76.0)	12 (80.0)	1 (25.0)	4 (44.4)	7 (87.5)	14 (82.4)	3 (50.0)
Monosensitized, *n* (%)^b^								
Only to *J. oxycedrus*	0 (0.0)	0 (0.0)	0 (0.0)	0 (0.0)	0 (0.0)	0 (0.0)	0 (0.0)	0 (0.0)
Only to *C. arizonica*	4 (5.5)	2 (8.7)	0 (0.0)	0 (0.0)	0 (0.0)	0 (0.0)	1 (6.3)	1 (20.0)
Only to Jun o 1	9 (12.3)	3 (13.0)	1 (7.1)	1 (50.0)	2 (33.3)	0 (0.0)	1 (6.3)	1 (20.0)
Only to Cup a 1	2 (2.7)	1 (4.3)	0 (0.0)	0 (0.0)	0 (0.0)	1 (14.3)	0 (0.0)	0 (0.0)

a Calculation performed in the subgroup of individuals sensitized to Cupressaceae and stratified by location.

b Calculation performed in the subgroup of individuals with Specific IgE to ≥1 species.

### 2.4 Enzyme-Linked immunoSorbent assay (ELISA) and competitive-ELISA

The procedures described below were performed following methodologies previously developed and reported by our group (17). Microlon, high binding Microtiter plates (Greiner bio-one), were coated ON with 1 µg of extract or 0.2 µg of purified allergen per well. After washing three times with PBS-T (PBS pH 7.4, 0.25% Tween 20), non-specific binding was blocked for 1 h with PBS-T containing 1% BSA. After three additional washing, plates were incubated ON with 1:5 diluted individual sera. After washing, plates were incubated with 1:2,000 horseradish peroxidase-conjugated secondary antibody: anti-human IgE (Southern Biotech). Finally, microplates were washed and incubated for 30 min with the substrate o-Phenylenediamine dihydrochloride (OPD) (Sigma-Aldrich). Reaction was stopped with 10% HCl and optical density (OD) was read at 492 nm. All the experiments were performed in duplicate. ODs from 11 negative sera were used to set the IgE cut-off (mean + 3 SD).

For competitive-ELISA microplates were coated and blocked as described before. Subsequently, 50 µL of serially diluted competitors (4-fold dilutions of *J. oxycedrus* and *C. arizonica* extracts, starting at 20 μg/mL, or ProGlycAn CCD inhibitor at 20 µg/mL) and 50 µL of diluted serum pool 1 (sera 10, 170, 176, 185 and 189) were added to the wells. The reaction was developed as described above. The positive control consisted of serum pool adsorbed with PBS-T containing 1% BSA without inhibitor.

### 2.5 Sodium dodecyl sulfate-polyacrylamide gel electrophoresis (SDS-PAGE) and western blot (WB)

The procedures described below were performed following methodologies previously developed and reported by our group (17). *J. oxycedrus* or *C. arizonica* pollen extracts were resolved under non reducing conditions in the presence of 10% SDS. The samples were separated in Any kD™ Mini-PROTEAN® TGX™ Precast Protein Gels (Bio-Rad), at 280 volts, using a Mini-PROTEAN II apparatus (Bio-Rad). For visualization, 5 µg of protein extract per lane were loaded when gels were stained with GelCode™ Blue Stain Reagent (Thermo Scientific), whereas 2 µg per lane were loaded for silver staining using the Pierce™ Silver Stain Kit (Thermo Scientific).

For WB, proteins were transferred onto a 0.45 µm nitrocellulose membrane (Bio-Rad) using a Transblot Semydry Electrophoresis transfer cell (Bio-Rad) at 20 volts for 30 min. The transferred membrane was blocked with PBS-T containing 5% BSA, for 60 min, and subsequently washed three times with PBS-T and incubated ON with 1:10 diluted individual sera at 4°C. After washing, the membranes were incubated with 1:2,000 horseradish peroxidase-conjugated mouse anti-human IgE (Southern Biotech) diluted in PBS-T containing 5% BSA for 1 h at room temperature. Protein-antibody binding was detected with ECL Prime detection reagent (Ge Healthcare Life Sciences) and chemiluminescence measured in a ChemiDoc imaging system (Bio-Rad). Precision Plus Protein WesternC Standards (Bio-Rad), previously incubated with Precision Protein StrepTactin-HRP Conjugate (Bio-Rad), were used to determine the relative molecular weights.

Alternatively, a WB inhibition assay was performed. For this experiment, proteins from *J. oxycedrus* and *C. arizonica* pollen extracts were immobilized on the solid phase, and serum pool 1 was used as the IgE source, previously incubated with 5 µg/mL of heterologous inhibitors (*J. oxycedrus* or *C. arizonica* pollen extracts), and the blots were subsequently revealed following the same detection procedure described above.

### 2.6 Two-dimensional electrophoresis and IgE-binding (2-DE)

The procedures described below were performed following methodologies previously developed and reported by our group (17). *J. oxycedrus* and *C. arizonica* pollen was extracted with PBS as described above. The supernatant was filtered through a 0.7 µm pore membrane (Sartorius Stedim Biotech), followed by two protein precipitations with 90% ammonium sulphate. Afterwards, the precipitate was resuspended in 0.5x Phosphate-Buffered Saline (PBS; 68.5 mM NaCl, 1.35 mM KCl, 5 mM Na_2_HPO_4_, and 0.9 mM KH_2_PO_4_), dialyzed against the same solution, and lyophilized until usage. Lyophilized samples were resolubilized with 2-D rehydration buffer containing 7 M urea, 2 M thiourea, 1% (w/v) ASB-14 detergent, 40 mM Tris base, and 0.001% bromophenol blue; before cleaning with ReadyPrepTM 2-D Cleanup Kit (Bio-Rad). Cleaned samples were resuspended in ReadyPrep 2-D starter kit rehydration/sample buffer containing 8 M urea, 2% CHAPS, 50 mM DTT, 0.2% Bio-Lyte 3/10 ampholyte and bromophenol blue. Protein concentration was determined using the RC DC protein assay kit (Bio-Rad).

Isoelectric focusing (IEF) was performed using 11 cm, pH 3–10 linear IPG strips (Bio-Rad), rehydrated with 185 μL of sample containing 180 μg of protein from *J. oxycedrus* or 75 μg from *C. arizonica*. Protein loading was normalized based on densitometric quantification of the five most abundant Jun o 1 and Cup a 1 spots in preliminary 2-DE analyses, in order to achieve comparable levels of group 1 allergen representation between extracts. This strategy was used to ensure that the most clinically relevant allergenic fraction was equivalent across samples, allowing differences in IgE-binding patterns outside group 1 allergens, to be assessed under standardized allergen exposure conditions. Proteins were focused at 50 μA/IPG strip for a total of 26.000 Vh at 20 °C using the PROTEAN i12 IEF System (Bio-Rad). After focusing, the strips were washed 10 min in equilibration buffer 1 containing 6 M urea, 20% v/v glycerol, 2% w/v SDS, 0.375 M Tris-HCl (pH 8.8) and 2% DTT, followed by equilibration buffer 2 containing 6 M urea, 20% v/v glycerol, 2% w/v SDS, 0.375 M Tris-HCl (pH 8.8) and 2.5% w/v iodoacetamide. Afterwards, IPG strips were washed with SDS-PAGE running buffer (25 mM Tris, 192 mM glycine, and 0.1% SDS) and applied onto the top of 12.5%, 11 cm, Criterion™ Precast Gels (Bio-Rad). Strips were covered with ReadyPrep Overlay Agarose (Bio-Rad) and electrophoresis performed at 200 V for 65 min. Gels were stained with GelCode Blue stain reagent (Life technologies). Precision Plus Protein Standard Plugs (Bio-Rad) were used to determine the molecular weights.

IgE-binding spots resolved by 2-DE were detected by WB, as previously described, using serum pool 2 (sera 1, 10, 33, 41, 68, 124, 170, 185, and 189; in Supplementary Table S1) and serum pool 3 (sera 51, 140, 141, 162, 173, and 180; in Supplementary Table S1).

### 2.7 Selection of specific IgE-binding spots and mass spectrometry analysis

The procedures described below were performed following methodologies previously developed and reported by our group (17). Coomassie stained and immunoblotted 2-D gels were compared using Image Lab image acquisition and analysis software (BioRad). Each immunoreactive spot was located on the image of the corresponding immunoblot and Coomassie stained gel. Spots that matched with similar molecular weight and IEP were further characterized by MS. The proteomic analysis was performed in the Proteomics Unit (CAI Biological Techniques) of Complutense University of Madrid.

Spots of interest were manually excised from gels, and in-gel reduced, alkylated and digested with trypsin, as described (20). Briefly, the samples were reduced with 10 mM DTT in 25 mM ammonium bicarbonate for 30 min at 56 °C and subsequently alkylated with 25 mM iodoacetamide in 25 mM ammonium bicarbonate for 15 min in the dark. Finally, samples were digested with 12.5 ng/μl sequencing grade trypsin (Roche Molecular Biochemicals) in 25 mM ammonium bicarbonate (pH 8.5) ON at 37 °C. After digestion, the supernatant was collected and 1 μl was spotted onto a MALDI target plate and allowed to air-dry at room temperature. Then, 0.6 μl of a 3 mg/mL of α-cyano-4-hydroxy-cinnamic acid matrix (Sigma) in 50% acetonitrile were added to the dried peptide digest spots and allowed again to air-dry at room temperature.

MALDI-TOF MS analyses and MS/MS sequencing analyses were performed in a 4,800 Plus Proteomics MALDI-TOF/TOF analyzer (AB Sciex). The MALDI-TOF/TOF operated in positive reflector mode with an accelerating voltage of 20,000 V. All mass spectra were calibrated internally using peptides from the auto digestion of trypsin. The MS spectra suitable precursor were selected for fragmentation analyses by CID (atmospheric gas was used) using a 1 kV ion reflector method and precursor mass Windows +/− 4 Da. The plate model & default calibration were optimized for the MS-MS spectra procesing.

For protein identification Uniprot data base with taxonomy restriction for “Viridiplantae” (13279282 sequences; 4758228666 residues), was searched using MASCOT 2.8 (https://www.matrixscience.com) through the software Protein Pilot 4.5 (ABSciex). Search parameters were: Carbamidomethyl Cystein as fixed modification and oxidized methionine as variable modification, Peptide mass tolerance: 80 ppm (Combined search), 1 missed trypsin cleavage site, MS-MS fragments tolerance, 0.3 Da. The parameters for the combined search (Peptide mass fingerprint plus MS-MS spectra) were the same described above. In all protein identification, the probability scores were greater than the score fixed by mascot as significant with a *p*-value minor than 0.05.

### 2.8 Statistical analysis

Descriptive statistics for the study population were calculated for the complete serum cohort, including mean age, standard deviation (SD), and percentages of male and female participants. Data distribution was assessed using the Kolmogorov–Smirnov normality test, which demonstrated that the study groups were not normally distributed. Therefore, non-parametric statistical tests were applied. Differences in IgE sensitization to *J. oxycedrus* and *C. arizonica* pollen extracts, as well as to their major allergens Jun o 1 and Cup a 1, were analyzed using the Kruskal–Wallis test followed by Dunn's multiple-comparison *post hoc* test. For ELISA-competition assays, homologous and heterologous inhibition experiments were performed using increasing concentrations of competitor extracts, as described above. Inhibition assays were independently repeated four times. Differences in inhibition percentages between competitors at each concentration were analyzed using multiple Mann–Whitney *U*-tests with false discovery rate (FDR) correction using the Benjamini, Krieger and Yekutieli method. Comparisons of monosensitization frequencies between paired Cupressaceae pollen species or molecular allergens were performed using an exact paired test based on the discordant monosensitized categories (equivalent to an exact McNemar/binomial test). As previous findings from our earlier study (17) suggested a higher frequency of monosensitization to *J. oxycedrus* and its major allergen Jun o 1, these predefined comparisons were evaluated using one-sided exact tests. Results were considered statistically significant when *p* < 0.05. All statistical analyses were performed using GraphPad Prism version 10 (GraphPad Software, San Diego, CA, USA).

## 3 Results

### 3.1 Protein pattern analysis of *J. oxycedrus* and *C. arizonica* extracts

**Figure 1:**
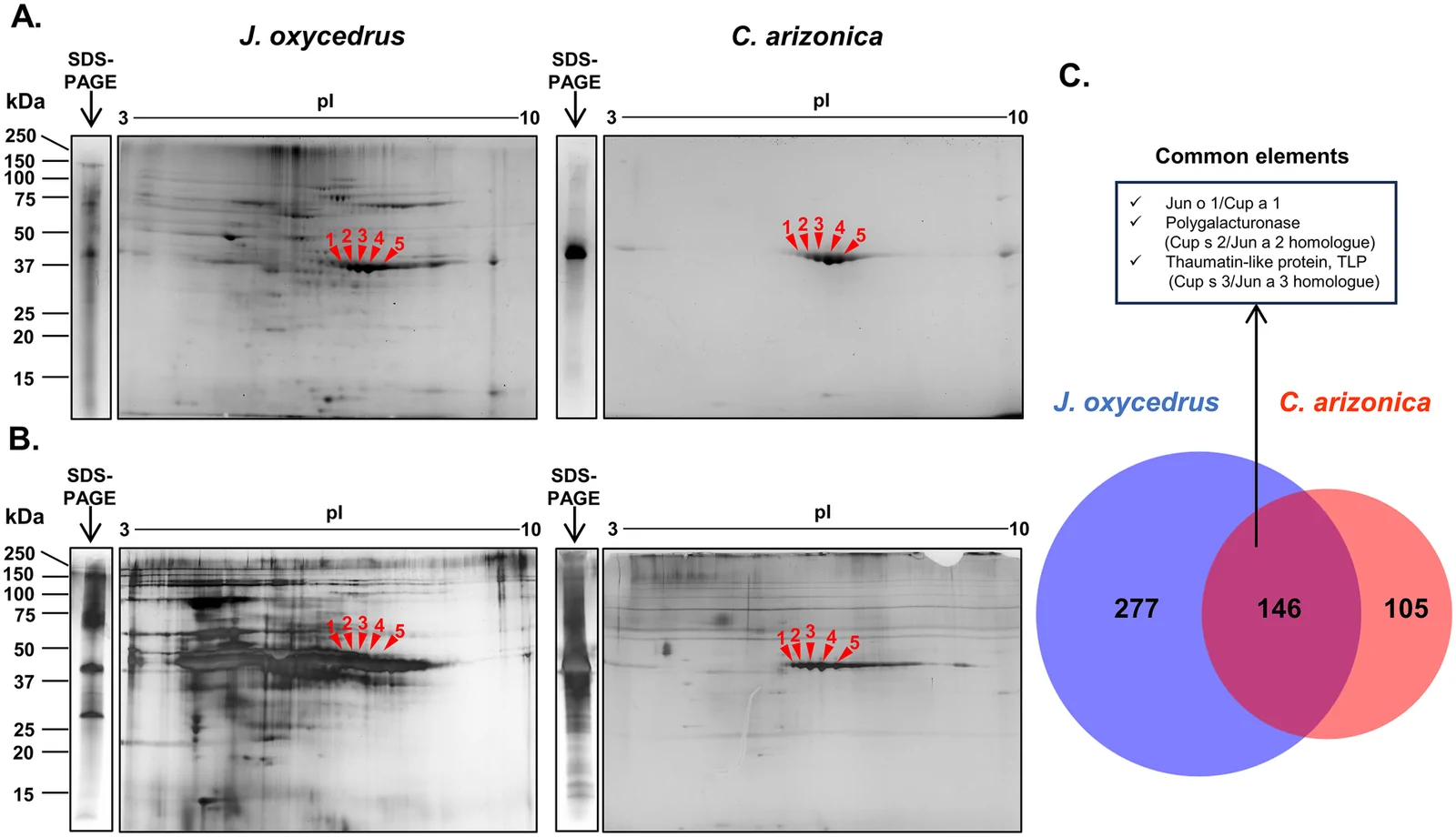
Characterization of protein content and IgE reactivity of *J. oxycedrus* and *C. arizonica* pollen extracts. **(A,B)** Proteins from *J. oxycedrus* and *C. arizonica* pollen extracts were separated by one-dimensional SDS-PAGE and 2D-electrophoresis, followed by Coomassie staining **(A)** or silver staining **(B)**. **(C)** Qualitative analysis by MS of the protein profiles shows 146 proteins common to both extracts, including the major allergens Jun o 1 and Cup a 1, as well as homologues of Cupressaceae allergens Cup s 2/Jun a 2 and Cup s 3/Jun a 3 (polygalacturonase and Thaumatin-like protein, TLP; respectively). Additionally, 277 proteins were unique to *J. oxycedrus* and 105 to *C. arizonica*.

Initial characterization of pollen extracts prepared from *J. oxycedrus* and *C. arizonica* by SDS-PAGE followed by Coomassie staining revealed clear qualitative differences between the two species. The *J. oxycedrus* extract displayed expected bands corresponding to Jun o 1, together with additional faintly stained components, whereas the *C. arizonica* extract was predominantly represented by a major band at approximately 42 kDa, consistent with Cup a 1 (Figures 1A,B). Due to the low intensity and poor resolution of single-dimensional bands, 2-DE was performed. Strips were loaded with 180 μg protein from *J. oxycedrus* or 75 μg from *C. arizonica*. These loading amounts were determined based on preliminary 2-DE analyses showing that Jun o 1 and Cup a 1 each appear as multiple isoforms distributed across several spots, as previously described (17). The five most intense spots corresponding to these allergens (arrows in Figures 1A,B) were used as reference features, and their combined densitometric signal was quantified in both species. Total protein loads were then adjusted to achieve comparable cumulative intensities of these group 1-derived spots. This normalization strategy ensured equivalent representation of the major allergenic fraction between species, thereby enabling a more robust comparison of IgE-binding patterns attributable to non-group 1 protein components.

The 2-DE gels obtained by Coomassie stain suggested a higher number of detectable protein spots in *J. oxycedrus*, whereas *C. arizonica* appears predominantly enriched in spots corresponding to Cup a 1 isoforms. Considering the limited sensitivity of Coomassie staining for low-abundance proteins, SDS-PAGE and 2-DE were repeated using silver staining to improve detection sensitivity. Under these conditions, a substantially increased number of protein bands and spots were detected in both extracts, revealing a more complex protein profile in *C. arizonica* than initially observed, and confirming the presence of additional low-abundance components not detectable under less sensitive staining conditions (Figures 1A,B).

To complement the electrophoretic analyses, qualitative MS analysis of *J. oxycedrus* and *C. arizonica* extracts (Figure 1C) identified 423 and 251 proteins, respectively. Of these, 146 were common to both species, including Jun o 1 and Cup a 1, while 277 were specific to *J. oxycedrus* and 105 to *C. arizonica*. Overall, 34.5% of the *J. oxycedrus*-derived proteins were shared with *C. arizonica*, and 58% of the *C. arizonica* proteins were represented in *J. oxycedrus*.

### 3.2 Study population

We examined 241 individuals aged 12–80 years (mean age 33 years, SD:15.2 years) with allergic rhinitis and/or conjunctivitis and evaluated their sensitization profiles using SPTs to three Cupressaceae species: *J. oxycedrus*, *C. arizonica*, and *C. sempervirens* (Table 1 and Supplementary Table S1). Overall, 84 individuals (35%) had positive SPT to at least 1 of the tested species. Among these, the frequency of sensitization to each individual species was very similar. When analysing cases of monosensitization, defined as a positive SPT to only one of the three species, monosensitization was higher for *J. oxycedrus* (8.3%) compared to *C. arizonica* (2.4%) and *C. sempervirens* (3.6%). However, these differences did not reach statistical significance in exact one-sided paired comparisons for the discordant monosensitized categories (*J. oxycedrus* vs. *C. arizonica*, *p* = 0.090; *J. oxycedrus* vs. *C. sempervirens*, *p* = 0.172). In this group, ELISA analysis showed that the proportion of individuals sensitized, either to at least one species or to each individual species, was consistently lower than that observed with SPT. By ELISA more individuals showed IgE reactivity to *C. arizonica* (69%) than to *J. oxycedrus* (55%). However, IgE reactivity analyses to purified allergens showed the opposite trend; a higher proportion of individuals were sensitized to Jun o 1 (79.8%) compared to Cup a 1 (71.4%). Monosensitization to Jun o 1 was more frequent than monosensitization to Cup a 1 (9 vs. 2 subjects; 12.3% vs. 2.7% among subjects with specific IgE to at least one Cupressaceae molecule/extract), and this difference was statistically significant (exact one-sided *p* = 0.033) (Table 1).

### 3.3 Cross-reactivity and western blot

**Figure 2:**
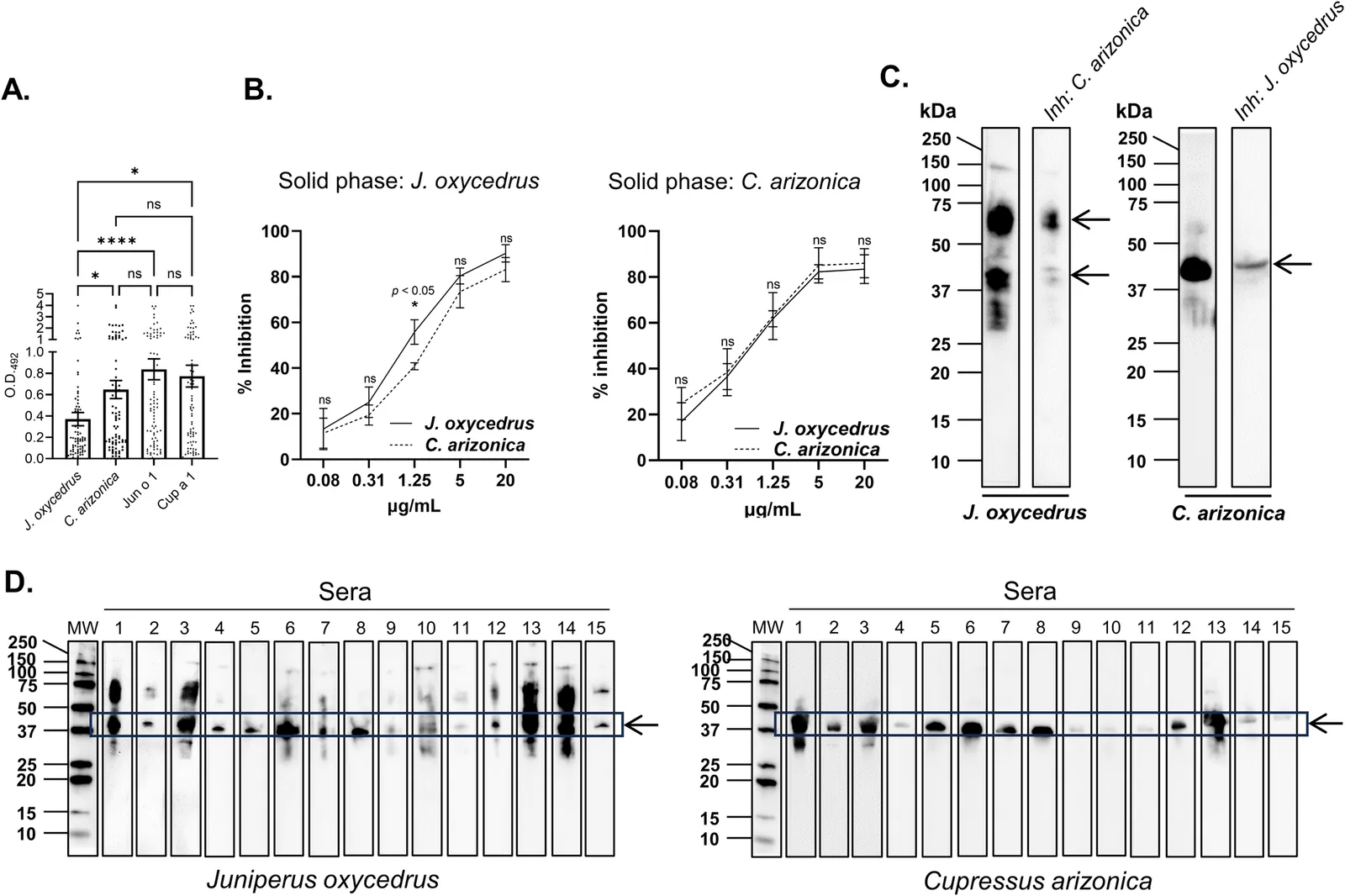
IgE reactivity and cross-inhibition between *J. oxycedrus* and *C. arizonica* pollen extracts. **(A)** IgE reactivity was measured by ELISA against *J. oxycedrus* and *C. arizonica* pollen extracts, Jun o 1, and Cup a 1. **(B)** Competitive ELISA performed using *J. oxycedrus* and *C. arizonica* pollen extracts as solid phases, assessing both homologous and heterologous inhibition. Statistically significant differences (*p*-value) at each concentration of inhibitor are displayed. **(C)** WB of *J. oxycedrus* and *C. arizonica* performed in parallel with heterologous inhibition assays. **(D)** WB using 15 individual sera against *J. oxycedrus* and *C. arizonica* extracts.

Specific IgE levels in the sera of patients with positive SPT to Cupressaceae, expressed as mean OD values ± SD, revealed that IgE reactivity to *J. oxycedrus* detected by ELISA was significantly lower than that to the *C. arizonica* extract or to the purified allergens (*p* < 0.05). In contrast, IgE levels to *C. arizonica*, Jun o 1, and Cup a 1 did not differ significantly from one another (Figure 2A) suggesting that IgE binding to *C. arizonica* is almost entirely driven by its major allergen.

Next, we conducted a competitive inhibition ELISA using serum pool 1 prepared from individual sera selected for their high levels of specific IgE against *J. oxycedrus*, including sera showing complete, partial, or no inhibition following CCD inhibition (Supplementary Figure S1), as well as high levels of specific IgE against *C. arizonica* and their major allergens, Jun o 1 and Cup a 1, and positive SPT responses to both species (Figure 2B). When *J. oxycedrus* extract was on the solid phase, homologous inhibition was slightly more potent than heterologous inhibition by *C. arizonica* extract, though statistically significant difference was only detected at 1.25 µg/mL inhibitor concentration. In contrast, when *C. arizonica* was used on the solid phase, both extracts induced comparable inhibition profiles across all concentrations (Figure 2B).

These asymmetric inhibition results suggest differences in the contribution of non-group-1 IgE-reactive components between the two species. Consistent with the proteomic findings (Figures 1A–C), *J. oxycedrus* appears to contain a greater representation of IgE-binding proteins outside the canonical group 1 allergens, which may contribute to the observed inhibition patterns. To confirm this, inhibition of WB using heterologous adsorption demonstrated that *C. arizonica* extract primarily inhibited the 41 kDa band corresponding to Jun o 1, with only minor effects on higher-molecular weight proteins, whereas *J. oxycedrus* almost completely inhibited the 42 kDa Cup a 1-related band in *C. arizonica* (Figure 2C). Furthermore, immunoblotting with sera from 15 randomly selected sensitized individuals (Figure 2D), confirmed that all sera recognized group-1 allergens in both pollen sources; however, *J. oxycedrus* exhibited a broader pattern of IgE-reactive bands, which may reflect a higher relative representation of non-group 1 allergenic components rather than their exclusive presence.

### 3.4 Two-dimensional electrophoresis and western blotting

To investigate IgE reactivity beyond the canonical group-1 allergens, we performed 2-DE followed by WB using serum pool 2. This pool was generated from individual sera selected based on a combined reactivity profile, including recognition of multiple IgE-binding bands in preliminary immunoblotting experiments, as well as positive skin SPT and specific IgE (based on ELISA) responses to both *J. oxycedrus* and *C. arizonica*. This selection strategy was designed to enrich for a broad spectrum of IgE specificities against Cupressaceae pollen components and to increase the likelihood of detecting non-group 1 IgE-binding proteins.

**Figure 3:**
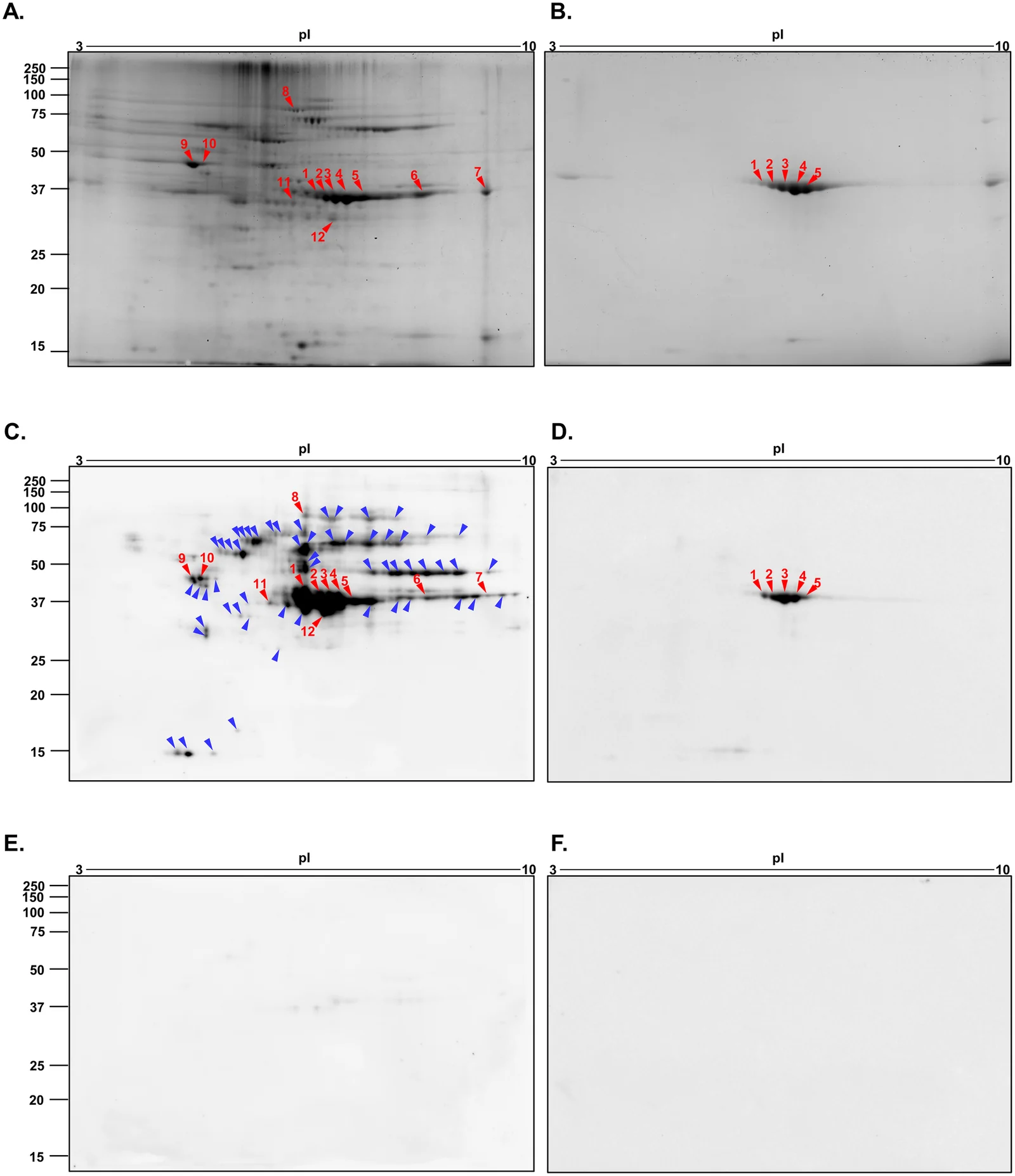
Two-dimensional electrophoresis and western blot analysis of *J. oxycedrus* and *C. arizonica* pollen extracts. **(A,B)** Proteins from *J. oxycedrus* **(A)** and *C. arizonica* **(B)** were separated by 2D-E and visualized by colloidal Coomassie staining. Western blotting was performed using a pool of sera from subjects with a combined reactivity profile, including recognition of multiple IgE-binding bands in preliminary immunoblotting experiments, as well as positive skin SPT and specific IgE (based on ELISA) responses to both *J. oxycedrus* and *C. arizonica* (shown in Supplementary Table S1) and a pool of SPT-negative sera. IgE-binding spots are shown for *J. oxycedrus* **(**C and E, respectively) and *C. arizonica* (D and F, respectively). Arrows indicate all IgE-reactive spots: red arrows (numbered 1–12) correspond to spots subsequently characterized by mass spectrometry, while blue arrows indicate spots that either had very low abundance in the Coomassie stain or were not detected by MS despite sufficient protein levels.

Using this strategy, *J. oxycedrus* showed at least 60 distinct IgE-binding spots (Figures 3A,C), compared with 5 spots detected in *C. arizonica* (Figures 3B,D). No IgE-binding spots were observed when pooled sera from SPT and ELISA-negative individuals were used (Figures 3E,F), confirming the specificity of the immunoreactive patterns.

MS-based identification was performed for the IgE-reactive spots from *J. oxycedrus*; however, due to low protein abundance and limited annotation of *J. oxycedrus* proteins in available databases, only spots 1 to 12 could be confidently identified (Table 2). Among the characterized IgE-reactive spots, group 1 allergens were confirmed as isoforms (spots 1–7). In addition, IgE-binding proteins included aconitate hydratase (spot 8), calreticulin (spots 9 and 10), aldose 1-epimerase (spot 11) and ∼29 kDa pectate lyase allergen, Jun o 1 (spot 12).

**Table 2:** Protein identification in *J. oxycedrus* and *C. arizonica* pollen extracts.

Spot no.	Accession number	Protein name	Function	Mascot protein score	Coverage (%)	Matched peptides/total peptides observed	Experimental MW (kDa)/pI	Allergen name	Homologue allergen
Spots from *J. oxycedrus* pollen extract 2-D electrophoresis									
1	Q93X51	Pectate lyase	Catalytic activity: Eliminative cleavage of (1->4)-*α*-D-galacturonan	95	18	6/70	41.0/6.5	Jun o 1	Cha o 1, Cry j 1, Cup a 1, Cup s 1, Jun a 1, Jun v 1
2				112	41	16/65	41.0/6.8		
3				142	42	21/65	41.0/7.2		
4				147	41	19/65	41.0/7.6		
5				76	37	13/65	41.0/7.9		
6				227	24	10/74	35.6/8.8		
7				54	13	4/72	36.3/10.0		
8	A0A7J6V8N4 · A0A7J6V8N4_THATH	Aconitate hydratase	Catalytic activity: citrate = D-threo-isocitrate	106	16	15/78	82.5/6.8	—	—
9	B8LP55 · B8LP55_PICSI	Calreticulin	Chaperone	330	19	13/75	45.6/5.0	—	—
10				153	12	8/71	45.6/5.2		
11	A0A0D6QXL2 · A0A0D6QXL2_ARACU	Aldose 1-epimerase	Catalytic activity: α-D-glucose = *β*-D-glucose	136	7	4/71	33.8/6.8	—	—
12	CAC48400.1	Pectate lyase	Catalytic activity: Eliminative cleavage of (1->4)-α-D-galacturonan	74	21	7/73	31.1/7.4	Jun o 1	Cha o 1, Cry j 1, Cup a 1, Cup s 1, Jun a 1, Jun v 1
13	A0A0D6R7B2 · A0A0D6R7B2_ARACU	Phosphopyruvate hydratase (Enolase)	Catalytic activity: 2-phospho-D-glycerate = phosphoenolpyruvate + H_2_O	491	32	15/75	45.1/6.4	—	
14	B6TIS2 · B6TIS2_MAIZE	Superoxide dismutase [Cu-Zn]	Catalytic activity: 2 superoxide + 2 H^+^ = H_2_O_2_ + O_2_	185	38	6/74	15.3/6.9	—	
Spots from *C. arizonica* pollen extract 2-D electrophoresis									
1	Q9SCG9	Pectate lyase	Catalytic activity: Eliminative cleavage of (1->4)-α-D-galacturonan	80	17	6/70	37.8/5.7	Cup a 1	Cha o 1, Cry j 1, Cup s 1, Jun a 1, Jun o 1, Jun v 1
2				151	28	13/70	37.8/5.8		
3				83	37	15/65	36.9/6.1		
4				100	37	16/65	36.4/6.2		
5				91	32	13/65	36.0/6.4		
6				121	41	20/65	36.0/6.6		
7				86	36	14/65	36.4/6.8		

MW, Molecular weight; pI, Isoelectric point.

To further characterize IgE-binding proteins potentially enriched in *J. oxycedrus*, we performed a separate 2-DE followed by WB using serum pool 3, previously selected to preferentially represent IgE responses associated with *J. oxycedrus* reactivity and non-group 1 components. This pool, enriched for individuals showing stronger serological and/or SPT reactivity to *J. oxycedrus* compared with *C. arizonica*, was used to complement earlier analyses based on group 1-normalized and broadly reactive serum pools.

**Figure 4:**
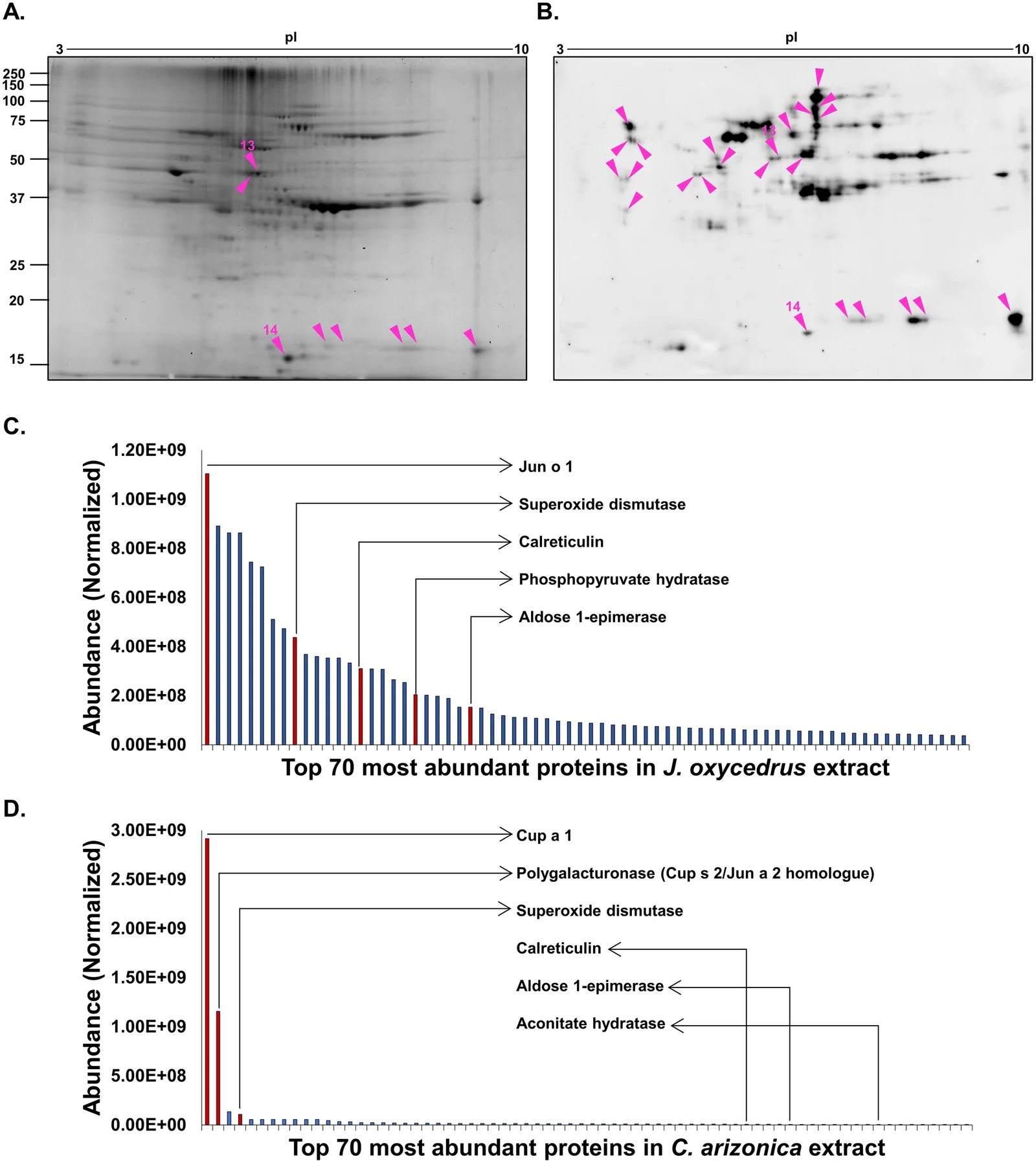
Western blot analysis of additional IgE-binding spots in *J. oxycedrus* and quantitative analysis of protein and IgE-binding composition in *J. oxycedrus* and *C. arizonica* pollen extracts. **(A,B)** 2-DE and IgE immunoblotting performed using a pool of sera enriched for individuals showing stronger serological and/or SPT reactivity to *J. oxycedrus* compared with *C. arizonica*. Additional IgE-binding spots, not detected in previous experiments, are indicated by magenta arrows in the Coomassie-stained gel **(A)** and the corresponding Western blot **(B)**. Spots numbered 13 and 14 represent two additional proteins subsequently characterized by mass spectrometry. Quantitative analysis of relative protein abundance is shown for *J. oxycedrus* **(C)** and *C. arizonica* **(D)**, with total proteins represented by blue bars and IgE-binding proteins highlighted in red. Polygalacturonase is also indicated as a homologue of Cup s 2/Jun a 2 allergens, identified by MS, although no IgE reactivity was detected with the serum pools used in this study.

The WB analysis revealed several spots that corresponded to previously detected IgE-binding proteins, as well as novel IgE-reactive spots (Figures 4A,B). Two of the novel IgE-reactive spots were characterized by MS (Table 2): phosphopyruvate hydratase or enolase (spot 13) and superoxide dismutase (spot 14). Additional IgE-binding spots were clearly visible on the immunoblots (Figure 4B) but were either not detected in the corresponding Coomassie-stained gels (Figure 4A) or could not be identified by MS.

Across both rounds of WB, the total IgE-binding spots detected may contain protein epitopes, CCDs, co-migrating glycoproteins, or combinations of these determinants.

### 3.5 Quantitative analysis of IgE-binding protein components in *J. oxycedrus* and *C. arizonica* extracts

To gain further insight into the relative abundance of the IgE-reactive proteins within the total protein content of *J. oxycedrus* and *C. arizonica* pollen extracts, we performed a quantitative analysis based on the MS results. For each species, the abundance of the characterized proteins was calculated relative to the total protein content identified in the extracts. The 70 most abundant proteins from each extract were plotted (Figures 4C,D), allowing a direct comparison of protein distribution and relative IgE-reactive protein abundance.

In both species, group 1 allergens represented the most abundant protein within the quantified proteome. In *J. oxycedrus*, however, several additional proteins also showed comparatively higher abundance and included four IgE-binding proteins identified by MS (superoxide dismutase, calreticulin, phosphopyruvate hydratase and aldose 1-epimerase). By contrast, in *C. arizonica*, aside from Cup a 1, only polygalacturonase (homologue of the Cupressaceae allergens Cup s 2/Jun a 2) was detected among the higher-abundance proteins (Figure 4D), whereas the remaining components showed a sharp decrease in relative abundance. Notably, four proteins (superoxide dismutase, calreticulin, aldose 1-epimerase, and aconitate hydratase) identified in *J. oxycedrus*, are present in *C. arizonica* and included in the top-70 proteins, but at lower relative abundance, suggesting that their contribution to overall IgE reactivity may be limited.

Overall, this quantitative assessment confirms that both pollen extracts harbor a complex protein composition, including all proteins corresponding to IgE-binding components identified in the 146 shared proteins by MS. The graphical representation highlights differences in the distribution of relatively abundant proteins, with *J. oxycedrus* showing a more even distribution of protein abundances compared with the more strongly group 1–dominated profile observed in *C. arizonica*.

## 4 Discussion

This study provides evidence that Cupressaceae pollen causing allergies exhibits a complex and heterogeneous IgE-binding composition, even among closely related species. Although group-1 allergens constitute the dominant allergenic fraction in both *J. oxycedrus* and *C. arizonica*, our findings suggest that sensitization to Cupressaceae cannot be attributed solely to group-1 allergens, contrary to prevailing assumptions. Through molecular, immunochemical, and proteomic approaches, we show that both species share a broadly similar IgE-binding repertoire, consistent with their close evolutionary relationship, but differ in the relative abundance and of several IgE-reactive proteins. In particular, *J. oxycedrus* displayed a less restricted protein distribution beyond the dominant group-1 fraction, including several IgE-binding proteins detected at substantially lower relative abundance in *C. arizonica* extracts. Importantly, the IgE reactivity associated with these components may result from recognition of protein epitopes, CCDs, or a combination of both molecular determinants, and therefore should not be interpreted exclusively as evidence of protein-driven sensitization. These results expand upon our previous characterization of Jun o 1 (17) where this allergen was identified as a structurally unique protein with distinct immunological behaviour, caused by oligomerization.

The IgE-reactive proteins identified include some previously associated with sensitization to fungi [calreticulin (21)] and plants [superoxide dismutase (22)], others found in diverse species [enolase, e.g., in (23–26)], in addition to aconitate hydratase and aldose 1-epimerase. Whereas the proteomic analysis also identified polygalacturonases and thaumatin-like proteins, which belong to allergen families already established in other Cupressaceae species, including Cup s 2 (27), Jun a 2 (28) and Cup s 3 (29) and Jun a 3 (30), respectively, these proteins were identified only within the pollen proteome by MS and were not detected among the IgE-reactive spots in the present study. Therefore, although they are recognized allergens in closely related Cupressaceae species, their IgE reactivity was not demonstrated here and, to our knowledge, they have not been formally characterized as allergens in *J. oxycedrus* or *C. arizonica*. As reviewed by Sharma and Vitte (31), the inference of allergen cross-reactivity requires evidence beyond shared membership in an allergen family. Accordingly, the occurrence of these allergen families in other sources does not necessarily indicate substantial structural similarity to infer shared allergenic properties or predict IgE cross-reactivity. Such potential cross-reactivity would require dedicated experimental evaluation.

The compositional differences observed between *J. oxycedrus* and *C. arizonica* may contribute to the different sensitization patterns detected in WB experiments. Similar observations have been reported in grasses (32) and Fagales (33, 34), where variation in allergen composition and relative allergen abundance have been associated with differences in sensitization profiles and species-specific immunological responses. Therefore, while *C. arizonica* largely represents a simplified allergenic model dominated by Cup a 1, *J. oxycedrus* combines the major group 1 allergen with additional molecular variants and a broader spectrum of IgE-binding proteins. Because these proteins were identified from IgE-reactive spots, the precise contribution of protein and carbohydrate determinants to the observed IgE recognition remains to be established.

Several lines of evidence support this interpretation. MS, silver staining and 2-DE results indicated that in *C. arizonica*, the relative abundance of Cup a 1 markedly exceeds that of any other protein in the extract. In contrast, although Jun o 1 is the most abundant protein in *J. oxycedrus*, many other proteins are present at intermediate levels rather than at low levels. Importantly, these differences in relative abundance of the total protein profile, are reflected in the IgE-binding protein content, which were detected in both pollen extracts but are generally more abundant in *J. oxycedrus* than in *C. arizonica*. However, the present study does not allow us to determine whether the corresponding IgE recognition is mediated predominantly by protein epitopes, CCDs, or both.

Cross-reactivity experiments were consistent with the overall differences in relative abundances of proteins and IgE-binding proteins between both species. *J. oxycedrus* showed a slightly greater inhibitory capacity than *C. arizonica*, particularly at lower inhibitor concentrations, suggesting a higher relative representation of relevant allergenic determinants within the extract. However, the strong inhibition achieved by both species and the convergence of inhibition curves at higher *C. arizonica* concentrations indicate extensive IgE-binding overlap. Accordingly, the modest asymmetry observed in inhibition profiles is more likely explained by differences in the relative abundance of shared IgE-reactive proteins than by the presence of species-specific IgE-binding proteins. Consistently, WB inhibition experiments showed that *C. arizonica* extract efficiently inhibited IgE binding to the ∼41 kDa Jun o 1 band in *J. oxycedrus*, while several additional IgE-reactive bands, including oligomeric Jun o 1 forms, remained only partially inhibited. Together, these findings support the notion that both species share their IgE-binding repertoire but differ in the relative contribution of individual IgE-binding components within the extract. Notably, a purely CCD-driven explanation does not completely account for the observed patterns. If the differences between species were exclusively attributable to shared carbohydrate determinants, a more symmetrical inhibition profile would be expected. Instead, the partial persistence of several *J. oxycedrus* IgE-reactive bands after inhibition with *C. arizonica* extract suggests that additional molecular determinants contribute to the observed reactivity. These determinants may involve protein epitopes, carbohydrate epitopes, or structurally integrated glycoprotein epitopes combining both components.

In our Spanish cohort 35% of patients were sensitized to at least one SPT-tested Cupressaceae species, comparable with previous studies (7, 8). As the study population consisted of individuals with allergic rhinitis and/or conjunctivitis who may be sensitized to multiple pollen sources, these findings reflect sensitization patterns rather than confirmed clinical allergy attributable to a specific Cupressaceae species. Sensitization frequencies were highly similar between *J. oxycedrus* and *C. arizonica*, supporting the extensive IgE-binding overlap expected between these closely related species. Nevertheless, monosensitization rates were slightly higher for *J. oxycedrus* by SPT, and Jun o 1 showed the highest monosensitization frequency in ELISA analyses. Although these differences were modest, they may reflect the contribution of additional IgE-reactive components in *J. oxycedrus*, including oligomeric Jun o 1 forms and proteins present at higher relative abundance compared with *C. arizonica*. Importantly, ELISA consistently detected lower sensitization rates than SPT, likely reflecting the reduced sensitivity of *in vitro* assays to reproduce the complexity and native presentation of IgE-binding determinants present in whole pollen extracts. In this context, SPT may provide a more physiologically representative assessment of sensitization, as it preserves the native allergenic environment and integrates the biological response to the complete extract. The persistence of comparable or slightly higher SPT reactivity to *J. oxycedrus*, despite its lower total group-1 content relative to Cup a 1 in *C. arizonica* extracts, further supports the potential relevance of additional IgE-reactive components in this species.

These findings have practical implications. From a molecular and immunological perspective, *J. oxycedrus* may represent a more regionally representative source of allergenic exposure in Mediterranean environments, as its pollen extract combines the canonical group-1 allergen with additional IgE-reactive components, including oligomeric Jun o 1 forms and other lower-abundance IgE-binding proteins that are also present in *C. arizonica*, but generally at lower relative abundance. In this context, although both species share a highly similar IgE-binding repertoire, diagnostic and immunotherapeutic approaches based exclusively on *C. arizonica* extracts or Cup a 1 may not fully reflect the relative distribution of allergenic components associated with sensitization patterns in Mediterranean populations. In this context a study conducted in France (35), using a non-Mediterranean Juniperus species, equivalent but not superior results compared to Cup a 1 were achieved, in terms of allergic responders. Thereby suggesting that approaches restricted to group 1 allergens and to non-native species may remain inherently limited; by contrast, our results raise the possibility that employing the autochthonous *J. oxycedrus* could help overcome some of these constraints.

The contribution of CCDs to the observed IgE-reactive patterns should also be considered. Although CCD-specific IgE is often regarded as a source of serological cross-reactivity (36), its biological relevance remains incompletely understood, particularly for glycosylated Cupressaceae allergens (37–39). Based on our previous findings in Cupressaceae allergy (17), the biological activity of native allergens appears to depend on the combined contribution of protein and glycan determinants rather than either component alone. However, these findings are specific to the group-1 allergens Jun o 1 and Cup a 1 and should not be extrapolated to imply that all CCD-mediated IgE binding is biologically or clinically relevant. Therefore, the broader spectrum of IgE-reactive components observed in *J. oxycedrus* cannot be attributed exclusively to either protein epitopes or CCDs, and further studies will be required to determine their relative contribution.

Several limitations of this study should be acknowledged. Although CCD-inhibition in ELISA experiments were performed on the representative subset of sera used to identify IgE-binding components, they were not performed on the WBs from which the IgE-binding proteins were identified. Consequently, the relative contribution of protein epitopes, CCDs, or both could not be established for each individual IgE-reactive spot. Therefore, the identified proteins are considered candidate IgE-binding proteins requiring further validation using purified native or recombinant non-glycosylated proteins. The newly identified IgE-binding proteins require confirmation using purified native or recombinant proteins, and validation in larger, and well-characterized cohorts of patients allergic to Cupressaceae, including clinical phenotypes beyond rhinitis. Future epidemiological studies should also include patients with diverse clinical manifestations beyond rhinitis to accurately determine prevalence and clinical relevance of sensitization to IgE-binding proteins in Cupressaceae. Moreover, the cross-sectional design precludes conclusions about primary sensitization, while the limited genomic and proteomic information available for *J. oxycedrus* may have constrained protein identification. Addressing these limitations will be necessary to validate our findings and support future advances in the understanding of Cupressaceae allergy. Furthermore, because most pollen-allergic patients are polysensitized and no allergen provocation tests or exposure-based assessments were performed, the clinical relevance of sensitization to individual Cupressaceae species could not be determined. Consequently, neither *J. oxycedrus* nor any other Cupressaceae species can be identified as the definitive cause of rhinitis and/or conjunctivitis symptoms in the studied patients. Our results therefore describe molecular and serological sensitization patterns rather than species-specific clinical allergy.

In conclusion, sensitization to the pollen of Cupressaceae in the Mediterranean area appears to involve recognition of a broad repertoire of molecular determinants beyond the dominant group-1 allergens. These determinants may include protein epitopes, CCDs, and combinations of both within glycosylated IgE-binding proteins. Extracts derived from pollen of different Cupressaceae species differ substantially in their relative abundance of IgE-binding content and may not adequately represent the accurate sensitizing profile of exposed populations. These findings encourage integrating region-specific allergen profiles into clinical practice, particularly in regions with diverse Cupressaceae flora.

## Data Availability

The raw data supporting the conclusions of this article will be made available by the authors, without undue reservation.
