# Supplementary material for: Proteomic profiling of Juniperus oxycedrus and Cupressus arizonica pollen reveals quantitative interspecies differences in the IgE-binding protein repertoire associated with Mediterranean Cupressaceae allergy
Source: Front Allergy. 2026 Aug 5;7:1885294. doi: 10.3389/falgy.2026.1885294 (PMC13487880; doi:10.3389/falgy.2026.1885294)
Supplement: Supplementary file 1 [file Table1.docx]

**Supplementary Figure legends**

**Supplemental Figure 1. Effect of CCD inhibition on serum IgE reactivity.** Individual sera composing Pool 1 were analyzed by ELISA against the *J. oxycedrus* pollen extract in the absence (dark blue bars) and presence (light blue bars) of ProGlycAn CCD inhibitor (1) at 20 µg/mL.

**Reference**

1.	Altmann F. Coping with cross-reactive carbohydrate determinants in allergy diagnosis. *Allergo J Int*. 2016;25(4):98-105. doi:10.1007/s40629-016-0115-3
