# Supplementary material for: Proteomic profiling of Juniperus oxycedrus and Cupressus arizonica pollen reveals quantitative interspecies differences in the IgE-binding protein repertoire associated with Mediterranean Cupressaceae allergy
Source: Front Allergy. 2026 Aug 5;7:1885294. doi: 10.3389/falgy.2026.1885294 (PMC13487880; doi:10.3389/falgy.2026.1885294)
Supplement: Supplementary file 2 [file Table2.docx]

**Table S1.** Demographic and sensitization profiles to *Juniperus oxycedrus*, *Cupressus arizonica*, *Cupressus sempervirens*, and the major allergens Jun o 1 and Cup a 1, of all patients with rhinoconjunctivitis considered in this study.

| **Location** | **Serum** | **Male** | **Age** | **Skin Prick Test (mm)** | | | **ELISA (OD_492_)*** | | | |
| --- | --- | --- | --- | --- | --- | --- | --- | --- | --- | --- |
|  |  |  |  | ***J. oxycedrus*** | ***C. arizonica*** | ***C. sempervirens*** | ***J. oxycedrus*** | ***C. arizonica*** | **Jun o 1** | **Cup a 1** |
| Salamanca | 1 | M | 30 | 0 | 0 | 5 | n/a | n/a | n/a | n/a |
|  | 2 | F | 47 | 0 | 6 | 6 | 0.029 | 0.157 | 0.051 | 0.244 |
|  | 3 | M | 65 | 4 | 4 | 0 | 0.044 | 0.025 | 0.001 | 0.048 |
|  | 4 | F | 19 | 4 | 6 | 4 | 0.003 | 0.032 | 0.038 | 0.011 |
|  | 5 | M | 16 | 6 | 6 | 6 | 0.549 | 1.647 | 1.658 | 2.050 |
|  | 6 | M | 59 | 0 | 0 | 0 | n/a | n/a | n/a | n/a |
|  | 7 | M | 21 | 0 | 0 | 0 | n/a | n/a | n/a | n/a |
|  | 8 | M | 46 | 8 | 7 | 6 | 0.063 | 0.081 | 0.265 | 0.082 |
|  | 9 | F | 46 | 6 | 8 | 6 | 0.160 | 0.644 | 0.462 | 0.886 |
|  | 10 | M | 54 | 0 | 5 | 6 | 0.320 | 0.475 | 0.309 | 0.321 |
|  | 11 | M | 57 | 0 | 0 | 0 | n/a | n/a | n/a | n/a |
|  | 12 | M | 41 | 8 | 8 | 6 | 0.248 | 0.490 | 0.522 | 0.475 |
|  | 13 | F | 24 | 0 | 0 | 0 | n/a | n/a | n/a | n/a |
|  | 14 | F | 39 | 0 | 0 | 0 | n/a | n/a | n/a | n/a |
|  | 15 | F | 28 | 0 | 0 | 0 | n/a | n/a | n/a | n/a |
|  | 16 | M | 35 | 4 | 6 | 8 | 0.090 | 0.224 | 0.196 | 0.234 |
|  | 17 | M | 26 | 6 | 6 | 5 | 0.582 | 1.152 | 1.335 | 1.181 |
|  | 18 | F | 53 | 6 | 6 | 9 | 0.079 | 0.437 | 0.433 | 0.699 |
|  | 19 | F | 60 | 6 | 5 | 6 | 0.250 | 0.593 | 0.766 | 0.822 |
|  | 20 | M | 40 | 6 | 5 | 6 | 0.175 | 0.304 | 0.484 | 0.465 |
|  | 21 | F | 52 | 0 | 0 | 0 | n/a | n/a | n/a | n/a |
|  | 22 | F | 24 | 0 | 0 | 0 | n/a | n/a | n/a | n/a |
|  | 23 | M | 35 | 5 | 6 | 6 | 0.484 | 1.055 | 1.275 | 1.179 |
|  | 24 | F | 22 | 0 | 0 | 0 | n/a | n/a | n/a | n/a |
|  | 25 | F | 40 | 0 | 0 | 0 | n/a | n/a | n/a | n/a |
|  | 26 | F | 51 | 8 | 6 | 6 | 0.183 | 0.283 | 0.606 | 0.340 |
|  | 27 | F | 51 | 6 | 9 | 8 | 0.126 | 0.425 | 0.545 | 0.713 |
|  | 28 | F | 16 | 5 | 6 | 4 | 0.025 | 0.179 | 0.241 | 0.237 |
|  | 29 | F | 43 | 6 | 5 | 6 | 0.614 | 1.219 | 1.527 | 1.606 |
|  | 30 | F | 27 | 0 | 0 | 0 | n/a | n/a | n/a | n/a |
|  | 31 | F | 23 | 0 | 0 | 0 | n/a | n/a | n/a | n/a |
|  | 32 | F | 23 | 0 | 0 | 0 | n/a | n/a | n/a | n/a |
|  | 33 | F | 15 | 5 | 5 | 0 | 0.191 | 0.172 | 0.404 | 0.204 |
|  | 34 | F | 19 | 5 | 6 | 6 | 0.919 | 1.330 | 2.520 | 1.977 |
|  | 35 | F | 30 | 6 | 4 | 6 | 0.069 | 0.046 | 0.145 | 0.000 |
|  | 36 | F | 28 | 6 | 7 | 6 | 0.380 | 0.397 | 0.980 | 0.653 |
|  | 37 | M | 53 | 0 | 0 | 0 | n/a | n/a | n/a | n/a |
|  | 38 | F | 48 | 0 | 0 | 0 | n/a | n/a | n/a | n/a |
|  | 39 | M | 22 | 0 | 0 | 0 | n/a | n/a | n/a | n/a |
|  | 40 | F | 18 | 0 | 0 | 0 | n/a | n/a | n/a | n/a |
|  | 41 | M | 21 | 6 | 0 | 6 | 0.068 | 0.145 | 0.112 | 0.068 |
|  | 42 | F | 12 | 0 | 0 | 0 | n/a | n/a | n/a | n/a |
|  | 43 | M | 16 | 0 | 0 | 0 | n/a | n/a | n/a | n/a |
|  | 44 | F | 53 | 0 | 0 | 0 | n/a | n/a | n/a | n/a |
|  | 45 | M | 42 | 6 | 6 | 6 | 0.444 | 0.685 | 0.991 | 0.792 |
| Albacete | 46 | F | 26 | 0 | 0 | 0 | n/a | n/a | n/a | n/a |
|  | 47 | F | 60 | 7 | 6 | 7 | 0.290 | 0.659 | 0.701 | 0.754 |
|  | 48 | M | 19 | 10 | 9 | 8 | 0.464 | 1.681 | 2.018 | 2.654 |
|  | 49 | F | 26 | 0 | 0 | 0 | n/a | n/a | n/a | n/a |
|  | 50 | F | 35 | 0 | 0 | 0 | n/a | n/a | n/a | n/a |
|  | 51 | M | 20 | 5 | 3 | 0 | 0.083 | 0.083 | 0.578 | 0.040 |
|  | 52 | F | 53 | 0 | 0 | 0 | n/a | n/a | n/a | n/a |
|  | 53 | M | 22 | 6 | 11 | 7 | 0.351 | 1.014 | 1.600 | 1.656 |
|  | 54 | F | 39 | 0 | 0 | 0 | n/a | n/a | n/a | n/a |
|  | 55 | M | 20 | 0 | 0 | 0 | n/a | n/a | n/a | n/a |
|  | 56 | F | 23 | 0 | 0 | 0 | n/a | n/a | n/a | n/a |
|  | 57 | F | 17 | 0 | 0 | 0 | n/a | n/a | n/a | n/a |
|  | 58 | F | 21 | 0 | 0 | 0 | n/a | n/a | n/a | n/a |
|  | 59 | F | 24 | 0 | 0 | 0 | n/a | n/a | n/a | n/a |
|  | 60 | F | 20 | 0 | 0 | 0 | n/a | n/a | n/a | n/a |
|  | 61 | M | 46 | 7 | 7 | 6 | 0.445 | 1.292 | 1.899 | 2.012 |
|  | 62 | F | 20 | 4 | 4 | 4 | 0.459 | 2.244 | 2.403 | 3.613 |
|  | 63 | M | 46 | 5 | 0 | 0 | 0.038 | 0.021 | 0.039 | 0.000 |
|  | 64 | M | 18 | 0 | 0 | 0 | n/a | n/a | n/a | n/a |
|  | 65 | F | 21 | 4 | 6 | 5 | 0.461 | 1.627 | 1.990 | 2.435 |
|  | 66 | M | 15 | 6 | 8 | 5 | 0.236 | 1.062 | 0.988 | 1.137 |
|  | 67 | M | 15 | 0 | 0 | 0 | n/a | n/a | n/a | n/a |
|  | 68 | F | 19 | 11 | 13 | 8 | 1.148 | 2.262 | 2.954 | 3.317 |
|  | 69 | F | 32 | 0 | 0 | 0 | n/a | n/a | n/a | n/a |
|  | 70 | M | 19 | 11 | 8 | 8 | 0.414 | 0.917 | 1.391 | 1.398 |
|  | 71 | M | 36 | 0 | 0 | 0 | n/a | n/a | n/a | n/a |
|  | 72 | M | 29 | 0 | 0 | 0 | n/a | n/a | n/a | n/a |
|  | 73 | F | 16 | 0 | 0 | 0 | n/a | n/a | n/a | n/a |
|  | 74 | M | 54 | 0 | 0 | 0 | n/a | n/a | n/a | n/a |
|  | 75 | M | 31 | 0 | 0 | 0 | n/a | n/a | n/a | n/a |
|  | 76 | F | 19 | 6 | 8 | 7 | 0.125 | 0.301 | 0.375 | 0.560 |
|  | 77 | F | 14 | 8 | 3 | 7 | 0.114 | 0.153 | 0.121 | 0.035 |
|  | 78 | F | 24 | 8 | 8 | 4 | 0.226 | 0.842 | 0.880 | 1.043 |
|  | 79 | M | 18 | 10 | 11 | 6 | 0.071 | 0.408 | 0.559 | 0.815 |
|  | 80 | F | 34 | 0 | 0 | 0 | n/a | n/a | n/a | n/a |
| León | 81 | M | 19 | 0 | 0 | 0 | n/a | n/a | n/a | n/a |
|  | 82 | F | 60 | 0 | 0 | 0 | n/a | n/a | n/a | n/a |
|  | 83 | M | 16 | 0 | 0 | 0 | n/a | n/a | n/a | n/a |
|  | 84 | F | 16 | 0 | 0 | 1 | n/a | n/a | n/a | n/a |
|  | 85 | F | 52 | 0 | 0 | 0 | n/a | n/a | n/a | n/a |
|  | 86 | F | 22 | 0 | 0 | 0 | n/a | n/a | n/a | n/a |
|  | 87 | M | 30 | 0 | 0 | 0 | n/a | n/a | n/a | n/a |
|  | 88 | M | 13 | 0 | 0 | 0 | n/a | n/a | n/a | n/a |
|  | 89 | F | 14 | 0 | 0 | 0 | n/a | n/a | n/a | n/a |
|  | 90 | F | 19 | 0 | 0 | 0 | n/a | n/a | n/a | n/a |
|  | 91 | F | 45 | 0 | 0 | 0 | n/a | n/a | n/a | n/a |
|  | 92 | M | 18 | 0 | 0 | 0 | n/a | n/a | n/a | n/a |
|  | 93 | F | 24 | 0 | 0 | 0 | n/a | n/a | n/a | n/a |
|  | 94 | F | 17 | 0 | 0 | 0 | n/a | n/a | n/a | n/a |
|  | 95 | F | 37 | 0 | 0 | 0 | n/a | n/a | n/a | n/a |
|  | 96 | M | 29 | 0 | 0 | 0 | n/a | n/a | n/a | n/a |
|  | 97 | M | 16 | 0 | 0 | 0 | n/a | n/a | n/a | n/a |
|  | 98 | M | 15 | 0 | 0 | 0 | n/a | n/a | n/a | n/a |
|  | 99 | F | 20 | 0 | 0 | 0 | n/a | n/a | n/a | n/a |
|  | 100 | F | 25 | 0 | 0 | 0 | n/a | n/a | n/a | n/a |
|  | 101 | M | 18 | 0 | 0 | 5 | n/a | n/a | n/a | n/a |
|  | 102 | M | 18 | 3 | 0 | 0 | 0.000 | 0.000 | 0.000 | 0.000 |
|  | 103 | F | 14 | 0 | 0 | 0 | n/a | n/a | n/a | n/a |
|  | 104 | F | 19 | 0 | 0 | 0 | n/a | n/a | n/a | n/a |
|  | 105 | F | 44 | 0 | 0 | 0 | n/a | n/a | n/a | n/a |
|  | 106 | M | 56 | 0 | 0 | 0 | n/a | n/a | n/a | n/a |
|  | 107 | M | 61 | 0 | 0 | 0 | n/a | n/a | n/a | n/a |
|  | 108 | M | 23 | 0 | 0 | 0 | n/a | n/a | n/a | n/a |
|  | 109 | F | 14 | 0 | 0 | 0 | n/a | n/a | n/a | n/a |
|  | 110 | M | 18 | 0 | 0 | 0 | n/a | n/a | n/a | n/a |
|  | 111 | M | 18 | 0 | 0 | 0 | n/a | n/a | n/a | n/a |
|  | 112 | F | 50 | 0 | 0 | 0 | n/a | n/a | n/a | n/a |
|  | 113 | F | 36 | 0 | 0 | 0 | n/a | n/a | n/a | n/a |
|  | 114 | M | 14 | 8 | 4 | 7 | 0.183 | 0.151 | 0.289 | 0.000 |
|  | 115 | M | 29 | 8 | 15 | 9 | 0.489 | 1.765 | 1.602 | 2.085 |
| Málaga | 116 | M | 28 | 0 | 0 | 0 | n/a | n/a | n/a | n/a |
|  | 117 | F | 36 | 0 | 0 | 0 | n/a | n/a | n/a | n/a |
|  | 118 | M | 35 | 5 | 5 | 6 | 0.069 | 0.159 | 0.256 | 0.235 |
|  | 119 | F | 58 | 0 | 0 | 0 | n/a | n/a | n/a | n/a |
|  | 120 | M | 16 | 0 | 0 | 0 | n/a | n/a | n/a | n/a |
|  | 121 | M | 17 | 0 | 0 | 0 | n/a | n/a | n/a | n/a |
|  | 122 | M | 15 | 0 | 0 | 0 | n/a | n/a | n/a | n/a |
|  | 123 | M | 43 | 0 | 0 | 0 | n/a | n/a | n/a | n/a |
|  | 124 | M | 15 | 9 | 9 | 11 | 0.810 | 1.389 | 1.123 | 1.438 |
|  | 125 | M | 31 | 0 | 0 | 0 | n/a | n/a | n/a | n/a |
|  | 126 | M | 26 | 0 | 0 | 0 | n/a | n/a | n/a | n/a |
|  | 127 | F | 37 | 6 | 7 | 7 | 0.789 | 2.266 | 3.223 | 3.431 |
|  | 128 | M | 24 | 0 | 0 | 0 | n/a | n/a | n/a | n/a |
|  | 129 | M | 55 | 0 | 0 | 0 | n/a | n/a | n/a | n/a |
|  | 130 | F | 18 | 0 | 0 | 0 | n/a | n/a | n/a | n/a |
|  | 131 | F | 56 | 0 | 0 | 0 | n/a | n/a | n/a | n/a |
|  | 132 | M | 39 | 0 | 0 | 0 | n/a | n/a | n/a | n/a |
|  | 133 | F | 25 | 0 | 0 | 0 | n/a | n/a | n/a | n/a |
|  | 134 | F | 39 | 0 | 0 | 0 | n/a | n/a | n/a | n/a |
|  | 135 | F | 29 | 5 | 10 | 5 | 2.438 | 2.309 | 0.451 | 0.502 |
|  | 136 | F | 65 | 0 | 0 | 0 | n/a | n/a | n/a | n/a |
|  | 137 | F | 45 | 4 | 0 | 0 | 0.040 | 0.051 | 0.042 | 0.000 |
|  | 138 | F | 19 | 0 | 0 | 6 | n/a | n/a | n/a | n/a |
|  | 139 | M | 17 | 0 | 0 | 0 | n/a | n/a | n/a | n/a |
|  | 140 | M | 41 | 6 | 6 | 0 | 0.070 | 0.095 | 0.189 | 0.065 |
|  | 141 | F | 52 | 9 | 0 | 0 | 0.051 | 0.068 | 0.051 | 0.093 |
|  | 142 | F | 16 | 0 | 0 | 0 | n/a | n/a | n/a | n/a |
|  | 143 | M | 15 | 0 | 0 | 0 | n/a | n/a | n/a | n/a |
|  | 144 | M | 17 | 0 | 0 | 0 | n/a | n/a | n/a | n/a |
|  | 145 | M | 51 | 0 | 0 | 0 | n/a | n/a | n/a | n/a |
|  | 146 | M | 14 | 0 | 0 | 0 | n/a | n/a | n/a | n/a |
|  | 147 | F | 14 | 0 | 0 | 0 | n/a | n/a | n/a | n/a |
|  | 148 | M | 53 | 0 | 0 | 0 | n/a | n/a | n/a | n/a |
|  | 149 | F | 28 | 0 | 0 | 0 | n/a | n/a | n/a | n/a |
|  | 150 | F | 44 | 0 | 0 | 0 | n/a | n/a | n/a | n/a |
|  | 151 | F | 55 | 0 | 0 | 0 | n/a | n/a | n/a | n/a |
|  | 152 | F | 25 | 0 | 5 | 0 | 0.166 | 0.140 | 0.124 | 0.030 |
|  | 153 | F | 19 | 0 | 0 | 0 | n/a | n/a | n/a | n/a |
|  | 154 | F | 23 | 0 | 0 | 0 | n/a | n/a | n/a | n/a |
|  | 155 | F | 17 | 0 | 0 | 0 | n/a | n/a | n/a | n/a |
|  | 156 | M | 56 | 0 | 0 | 0 | n/a | n/a | n/a | n/a |
|  | 157 | F | 29 | 0 | 0 | 0 | n/a | n/a | n/a | n/a |
|  | 158 | M | 18 | 0 | 0 | 0 | n/a | n/a | n/a | n/a |
|  | 159 | F | 40 | 0 | 0 | 0 | n/a | n/a | n/a | n/a |
|  | 160 | M | 31 | 0 | 0 | 0 | n/a | n/a | n/a | n/a |
| Sevilla | 161 | M | 54 | 0 | 0 | 0 | n/a | n/a | n/a | n/a |
|  | 162 | F | 16 | 5 | 0 | 0 | 0.067 | 0.046 | 0.073 | 0.000 |
|  | 163 | F | 58 | 0 | 0 | 0 | n/a | n/a | n/a | n/a |
|  | 164 | F | 26 | 0 | 0 | 0 | n/a | n/a | n/a | n/a |
|  | 165 | F | 34 | 0 | 0 | 0 | n/a | n/a | n/a | n/a |
|  | 166 | M | 67 | 0 | 0 | 0 | n/a | n/a | n/a | n/a |
|  | 167 | M | 16 | 0 | 0 | 0 | n/a | n/a | n/a | n/a |
|  | 168 | F | 39 | 0 | 0 | 0 | n/a | n/a | n/a | n/a |
|  | 169 | M | 37 | 0 | 0 | 0 | n/a | n/a | n/a | n/a |
|  | 170 | F | 52 | 2 | 3 | 2 | 1.563 | 0.643 | 1.573 | 0.449 |
|  | 171 | M | 20 | 0 | 0 | 0 | n/a | n/a | n/a | n/a |
|  | 172 | M | 59 | 0 | 0 | 0 | n/a | n/a | n/a | n/a |
|  | 173 | M | 16 | 3 | 0 | 0 | 1.054 | 0.635 | 1.637 | 0.585 |
|  | 174 | M | 25 | 2 | 0 | 0 | 0.010 | 0.000 | 0.000 | 0.037 |
|  | 175 | M | 16 | 2 | 0 | 2 | 0.017 | 0.024 | 0.085 | 0.000 |
|  | 176 | M | 47 | 5 | 7 | 6 | 0.519 | 0.579 | 0.888 | 0.725 |
|  | 177 | F | 65 | 0 | 0 | 0 | n/a | n/a | n/a | n/a |
|  | 178 | F | 49 | 0 | 0 | 0 | n/a | n/a | n/a | n/a |
|  | 179 | F | 23 | 0 | 0 | 0 | n/a | n/a | n/a | n/a |
|  | 180 | M | 17 | 3 | 0 | 0 | 1.111 | 1.167 | 1.883 | 1.127 |
|  | 181 | M | 46 | 0 | 0 | 0 | n/a | n/a | n/a | n/a |
|  | 182 | M | 34 | 3 | 2 | 6 | 0.141 | 0.162 | 0.145 | 0.157 |
|  | 183 | F | 40 | 0 | 0 | 0 | n/a | n/a | n/a | n/a |
|  | 184 | M | 33 | 0 | 0 | 0 | n/a | n/a | n/a | n/a |
|  | 185 | M | 35 | 4 | 6 | 5 | 0.672 | 1.212 | 1.342 | 1.372 |
|  | 186 | F | 29 | 0 | 0 | 0 | n/a | n/a | n/a | n/a |
|  | 187 | F | 45 | 0 | 0 | 0 | n/a | n/a | n/a | n/a |
|  | 188 | M | 18 | 4 | 4 | 5 | 0.049 | 0.068 | 0.127 | 0.226 |
| Madrid | 190 | F | 21 | 4 | 9 | 4 | 3.975 | 3.989 | 3.727 | 3.923 |
|  | 191 | F | 26 | 0 | 4 | 4 | 0.111 | 0.107 | 0.088 | 0.052 |
|  | 192 | M | 34 | 0 | 0 | 0 | n/a | n/a | n/a | n/a |
|  | 193 | M | 28 | 0 | 0 | 0 | n/a | n/a | n/a | n/a |
|  | 194 | M | 48 | 4 | 8 | 6 | 1.803 | 2.213 | 3.946 | 2.385 |
|  | 195 | F | 34 | 6 | 10 | 7 | 0.187 | 0.763 | 1.183 | 1.013 |
|  | 196 | M | 32 | 0 | 0 | 0 | n/a | n/a | n/a | n/a |
|  | 197 | M | 36 | 6 | 9 | 5 | 0.561 | 0.690 | 0.748 | 0.699 |
|  | 198 | M | 24 | 7 | 6 | 5 | 0.196 | 0.361 | 0.487 | 0.397 |
|  | 199 | M | 16 | 4 | 4 | 3 | 0.161 | 0.204 | 0.170 | 0.145 |
|  | 200 | F | 48 | 0 | 0 | 0 | n/a | n/a | n/a | n/a |
|  | 201 | F | 61 | 0 | 0 | 0 | n/a | n/a | n/a | n/a |
|  | 202 | F | 33 | 0 | 0 | 0 | n/a | n/a | n/a | n/a |
|  | 203 | M | 40 | 7 | 8 | 7 | 0.108 | 0.380 | 0.518 | 0.481 |
|  | 204 | M | n/p | 4 | 8 | 4 | 0.057 | 0.194 | 0.345 | 0.281 |
|  | 205 | M | 31 | 4 | 6 | 5 | 1.552 | 3.810 | 3.946 | 3.923 |
|  | 206 | M | 31 | 5 | 8 | 9 | 0.630 | 1.107 | 1.353 | 1.211 |
|  | 207 | F | 51 | 4 | 4 | 4 | 0.200 | 0.209 | 0.307 | 0.124 |
|  | 208 | M | 42 | 9 | 4 | 4 | 0.115 | 0.133 | 0.470 | 0.152 |
|  | 209 | M | 43 | 0 | 0 | 0 | n/a | n/a | n/a | n/a |
|  | 210 | M | 17 | 7 | 8 | 7 | 0.011 | 0.063 | 0.110 | 0.000 |
|  | 211 | M | 27 | 0 | 0 | 0 | n/a | n/a | n/a | n/a |
|  | 212 | M | 58 | 6 | 0 | 5 | 0.000 | 0.000 | 0.011 | 0.089 |
|  | 213 | F | 32 | 0 | 0 | 0 | n/a | n/a | n/a | n/a |
|  | 214 | F | 41 | 5 | 7 | 6 | 0.301 | 0.623 | 0.906 | 0.738 |
|  | 215 | M | 24 | 5 | 6 | 5 | 0.166 | 0.373 | 0.720 | 0.416 |
| Barcelona | 216 | F | 65 | 5 | 7 | 5 | 0.000 | 0.049 | 0.226 | 0.064 |
|  | 217 | F | 65 | 5 | 4 | 4 | 0.000 | 0.000 | 0.000 | 0.000 |
|  | 218 | M | 19 | 0 | 0 | 0 | n/a | n/a | n/a | n/a |
|  | 219 | F | 28 | 0 | 0 | 0 | n/a | n/a | n/a | n/a |
|  | 220 | F | 61 | 0 | 0 | 0 | n/a | n/a | n/a | n/a |
|  | 221 | F | 72 | 0 | 0 | 0 | n/a | n/a | n/a | n/a |
|  | 222 | F | 31 | 0 | 0 | 0 | n/a | n/a | n/a | n/a |
|  | 223 | F | 36 | 0 | 0 | 0 | n/a | n/a | n/a | n/a |
|  | 224 | F | 69 | 0 | 0 | 0 | n/a | n/a | n/a | n/a |
|  | 225 | F | 73 | 0 | 0 | 0 | n/a | n/a | n/a | n/a |
|  | 226 | M | 29 | 7 | 10 | 6 | 0.194 | 0.430 | 0.868 | 0.539 |
|  | 227 | F | 45 | 4 | 9 | 4 | 0.000 | 0.110 | 0.092 | 0.050 |
|  | 228 | F | 47 | 0 | 0 | 0 | n/a | n/a | n/a | n/a |
|  | 229 | F | 18 | 0 | 0 | 0 | n/a | n/a | n/a | n/a |
|  | 230 | M | 17 | 0 | 0 | 0 | n/a | n/a | n/a | n/a |
|  | 231 | M | 28 | 0 | 7 | 6 | 0.038 | 0.170 | 0.266 | 0.271 |
|  | 232 | F | 47 | 0 | 0 | 0 | n/a | n/a | n/a | n/a |
|  | 233 | F | 44 | 0 | 0 | 0 | n/a | n/a | n/a | n/a |
|  | 234 | M | 42 | 0 | 0 | 0 | n/a | n/a | n/a | n/a |
|  | 235 | F | 41 | 0 | 0 | 0 | n/a | n/a | n/a | n/a |
|  | 236 | M | 27 | 0 | 0 | 0 | n/a | n/a | n/a | n/a |
|  | 237 | F | 35 | 0 | 0 | 0 | n/a | n/a | n/a | n/a |
|  | 238 | M | 54 | 0 | 0 | 0 | n/a | n/a | n/a | n/a |
|  | 239 | F | 30 | 0 | 0 | 0 | n/a | n/a | n/a | n/a |
|  | 240 | F | 16 | 0 | 0 | 0 | n/a | n/a | n/a | n/a |
|  | 241 | M | 28 | 0 | 9 | 5 | 0.087 | 0.157 | 0.558 | 0.250 |

* ELISA was performed for patients who had positive SPT to at least one of the three cypress species analysed*: J. oxycedrus*, *C. arizonica* and *C. sempervirens*

n/a: not analysed

n/p: not provided
